# Dioxin Induces Genomic Instability in Mouse Embryonic Fibroblasts

**DOI:** 10.1371/journal.pone.0037895

**Published:** 2012-05-29

**Authors:** Merja Korkalainen, Katriina Huumonen, Jonne Naarala, Matti Viluksela, Jukka Juutilainen

**Affiliations:** 1 Department of Environmental Health, National Institute for Health and Welfare, Kuopio, Finland; 2 Department of Environmental Science, University of Eastern Finland, Kuopio, Finland; University of Saarland Medical School, Germany

## Abstract

Ionizing radiation and certain other exposures have been shown to induce genomic instability (GI), i.e., delayed genetic damage observed many cell generations later in the progeny of the exposed cells. The aim of this study was to investigate induction of GI by a nongenotoxic carcinogen, 2,3,7,8-tetrachlorodibenzo-*p*-dioxin (TCDD). Mouse embryonic fibroblasts (C3H10T1/2) were exposed to 1, 10 or 100 nM TCDD for 2 days. Micronuclei (MN) and expression of selected cancer-related genes were assayed both immediately and at a delayed point in time (8 days). For comparison, similar experiments were done with cadmium, a known genotoxic agent. TCDD treatment induced an elevated frequency of MN at 8 days, but not directly after the exposure. TCDD-induced alterations in gene expression were also mostly delayed, with more changes observed at 8 days than at 2 days. Exposure to cadmium produced an opposite pattern of responses, with pronounced effects immediately after exposure but no increase in MN and few gene expression changes at 8 days. Although all responses to TCDD alone were delayed, menadione-induced DNA damage (measured by the Comet assay), was found to be increased directly after a 2-day TCDD exposure, indicating that the stability of the genome was compromised already at this time point. The results suggested a flat dose-response relationship consistent with dose-response data reported for radiation-induced GI. These findings indicate that TCDD, although not directly genotoxic, induces GI, which is associated with impaired DNA damage response.

## Introduction

Genomic instability (GI) is defined as an increased rate of acquisition of alterations in the genome [1]. GI can be observed many cell generations later in the progeny of exposed cells as delayed damage, e.g., chromosomal aberrations, mutations, micronuclei or apoptosis. Exposure to ionizing radiation is the best-known inducer of GI, but also chemical exposures can lead to GI, even though the data are limited [2]–[4]. GI is thought to be a driving force of carcinogenesis in both radiation- and chemical-induced cancer [5]. A key question for assessing the importance of GI in carcinogenesis is whether also “non-genotoxic” carcinogens (agents that do not cause genetic damage in traditional short-term tests) can induce GI.

2,3,7,8-Tetrachlorodibenzo-*p*-dioxin (TCDD) is classified as a group I carcinogen by the International Agency for Research on Cancer [6], but its carcinogenicity is not mediated by direct genotoxic effects. Based on evidence from animal experiments TCDD is a potent tumor promoter, but the tumor-initiating activity is either lacking or the response is weak [6], [7]. In this study, TCDD was chosen to investigate induction of GI as an agent that is not directly genotoxic. In general, TCDD is a model compound for dioxins, a group of wide-spread, persistent and highly toxic environmental contaminants. In experimental animals, TCDD evokes a wide range of biological and toxic effects, including reproductive and developmental defects, immunotoxicity, endocrine alterations, thymus atrophy, wasting syndrome, liver toxicity and cancer [8]–[11]. Practically all of these effects are mediated via the aryl hydrocarbon receptor (AHR), which, upon binding to TCDD, translocates into the nucleus, heterodimerizes with AHR nuclear translocator (ARNT) and binds to dioxin-responsive elements in DNA [12]. The best-known effect is the activation of genes for xenobiotic metabolism, such as CYP1A1, but otherwise the TCDD-induced mechanisms in other responses are still largely unknown. The ability of TCDD to induce GI has not been previously investigated.

The aim of the present study was to investigate TCDD-induced GI in mouse embryonic fibroblasts, which have been utilized earlier to study GI [13]. For comparison, the cells were exposed also to cadmium chloride, a known genotoxic compound. GI was assayed by measuring delayed induction of micronuclei (MN) several cell generations after exposure. MN were used also to assess immediate genetic damage after exposure. As another indicator of decreased stability of the genome, we tested whether pre-exposure to TCDD modifies menadione-induced DNA damage and DNA repair. Menadione was selected, as we have previously shown that a nongenotoxic agent (extremely low frequency magnetic field) alters cellular responses to a subsequent exposure to menadione [14], [15]. In addition, expression of cancer-related genes was studied, because altered expression of specific genes might indicate mechanisms involved in maintaining the unstable phenotype over multiple cell generations. Also, Fält et al. [16] found changes in the gene expression pattern of irradiated T-lymphocyte clones cultured for multiple generations after exposure to ionizing radiation.

## Results

For every assay, mouse embryo fibroblasts were exposed to TCDD for 2 days, after which the cells were cultured without exposure for 6 or 13 more days (time points 8 and 15 days) in order to measure both direct and delayed responses in the progeny of the exposed cells. Different TCDD concentrations (1–100 nM) were used in the micronucleus and Comet assays, and 10 nM TCDD was chosen for the gene expression analysis, because it gave maximal response in MN frequency. Cadmium concentration was 1 µM in all experiments, as preliminary studies showed that it was genotoxic at this level. Cell proliferation was measured after exposure to confirm that the doses are not cytotoxic. Even the highest dose of TCDD (100 nM) did not affect the proliferation rate (data not shown).

**Figure 1:**
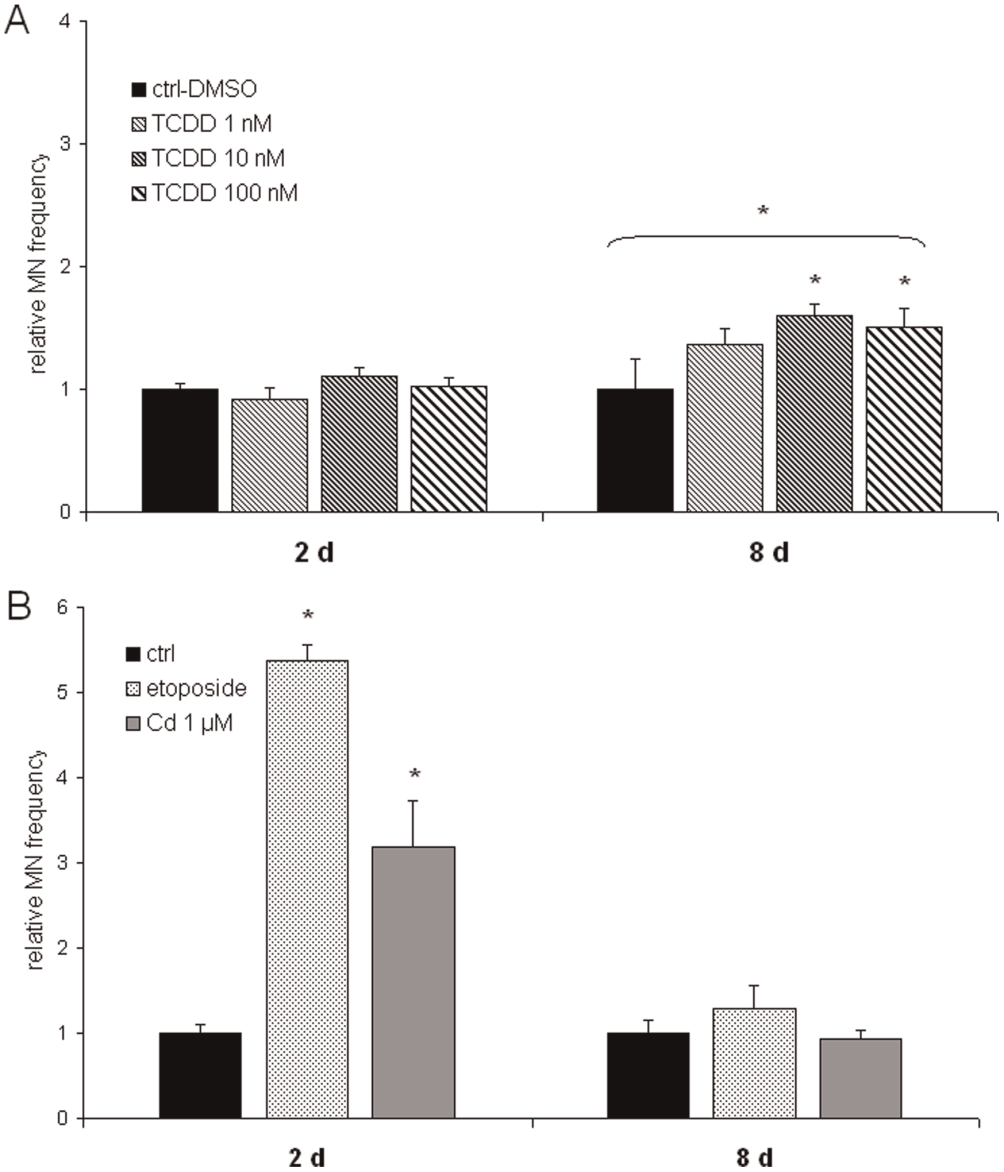
Induction of micronuclei. The effect of 1, 10 or 100 nM TCDD (A) and 1 µM cadmium (B) on relative micronucleus (MN) frequency in mouse embryonal fibroblasts was determined immediately after exposure for 2 days and at the end of 6 days of recovery without exposure. Etoposide (0.025 µg/µl) was used as a positive control. Each column represents mean ± SE of two replicates in 3–4 independent experiments. Statistically significant differences are indicated by asterisks (*).

TCDD induced an elevated frequency of MN at 8 days, but not directly after the exposure at 2 days (Fig. 1a). ANOVA analysis of this delayed effect showed a significant overall effect (p = 0.02), and the post tests indicated a significant trend (p = 0.007), and significant effects (p<0.05) at 10 and 100 nM TCDD. The differences between the three doses were small, with no clear dose-dependence. Cadmium, however, induced elevated MN frequency only directly (p = 0.004); no delayed effects were observed (Fig. 1b). The positive control, etoposide, produced the highest increase in MN frequency (5.3-fold compared to controls, p = 0.008).

**Figure 2:**
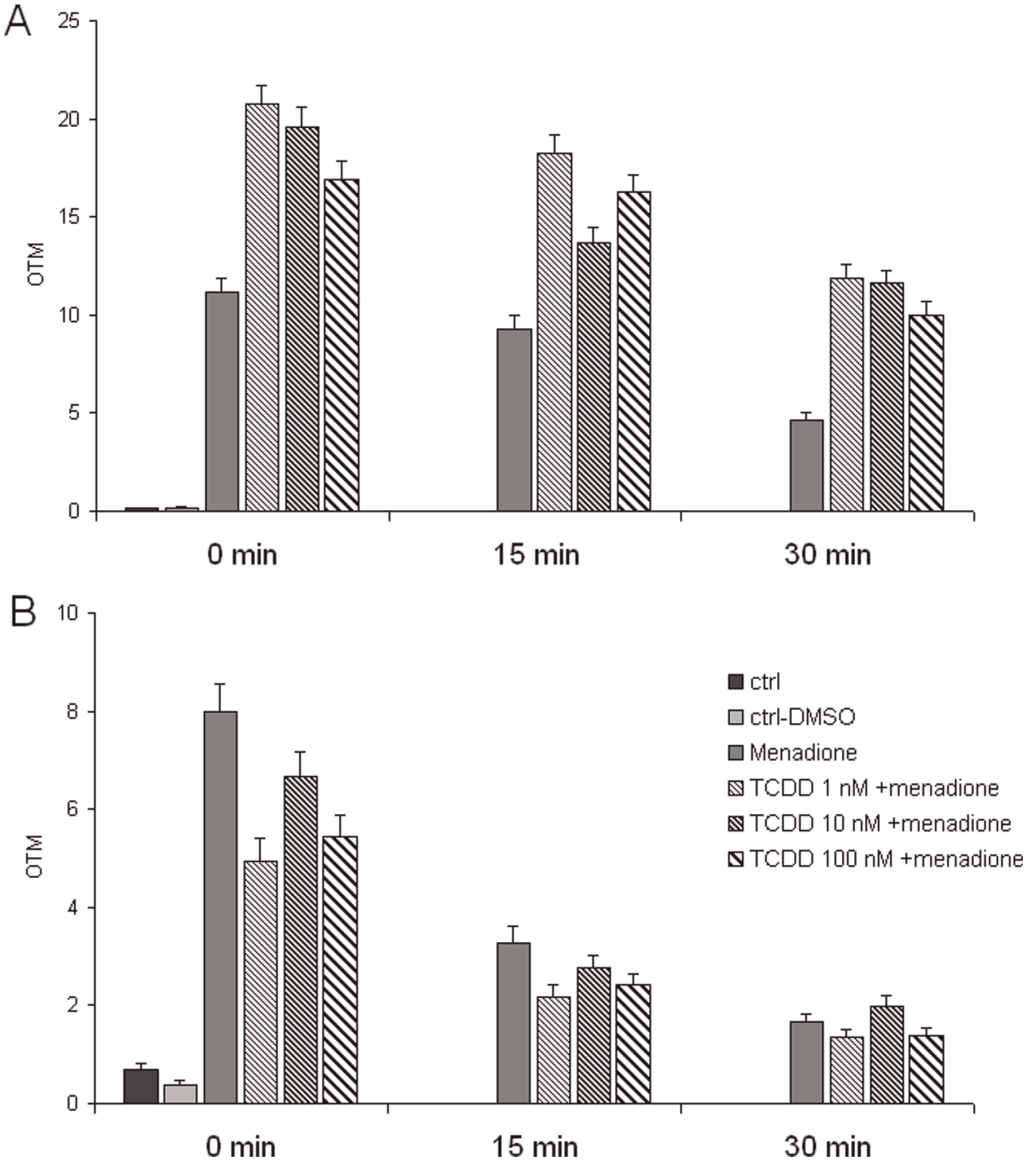
Effect of TCDD pretreatment on menadione-induced DNA damage and its repair. Comet tail moments were analyzed after TCDD exposure (1, 10 or 100 nM) for 2 days (A) and at the end of 6 days recovery time without exposure (B). After menadione treatment (40 µM) for one hour, cells were allowed to repair menadione-induced DNA damage for 0, 15, or 30 min. Each column represents mean ± SE of 400 Olive tail moments (OTM) in 4 independent experiments (A) or mean ± SE of 300 tail moments of 3 independent experiments (B). The effect of TCDD, tested over all TCDD doses and all three time points, was significant (p = 0.0009) when measured immediately after TCDD exposure, but not at 6 days after the end of exposure. The effect of menadione was significant (p<0.0001) in both cases.

**Figure 3:**
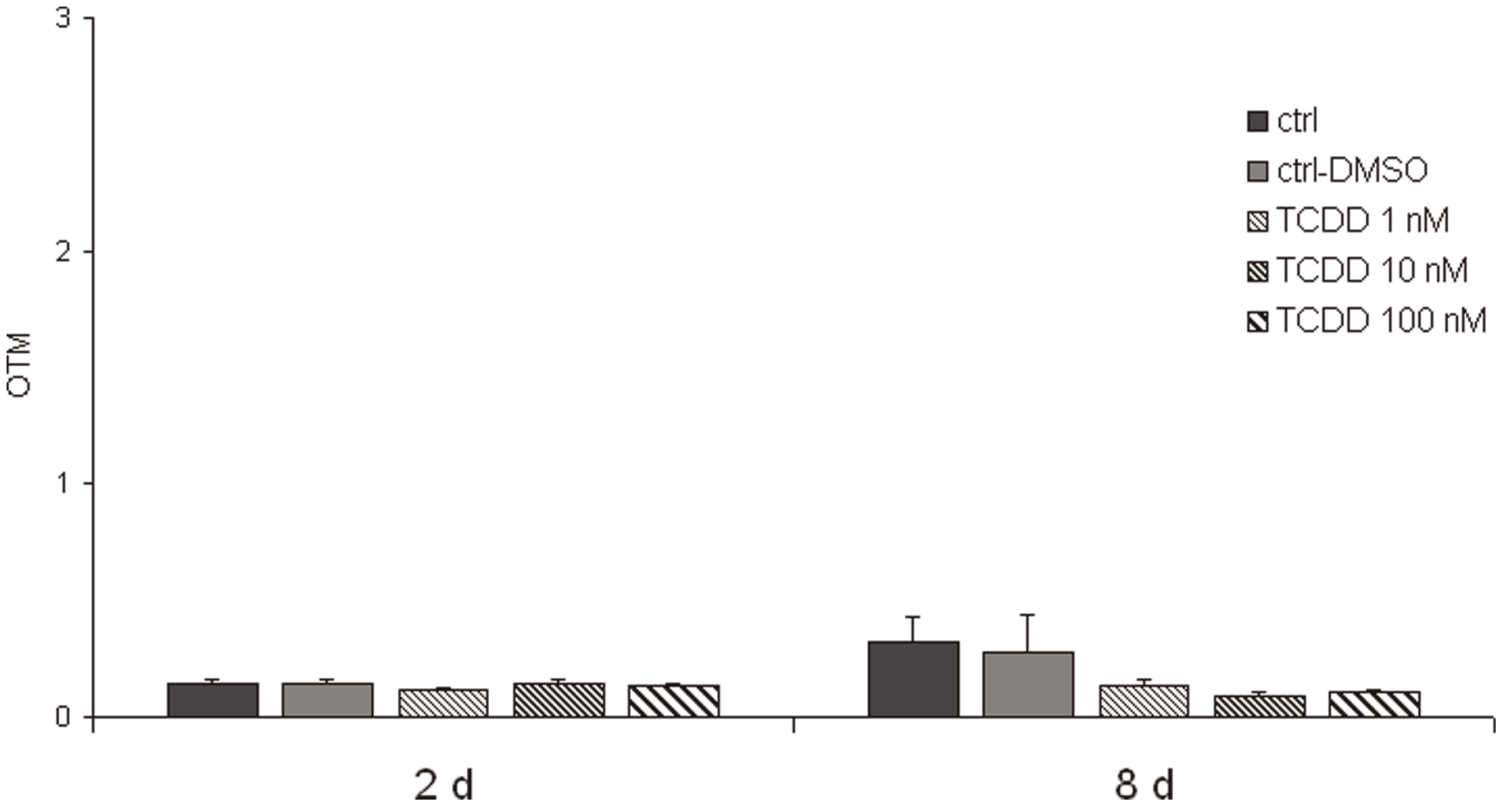
Comet assay after TCDD treatment. Comet tail moments were analyzed after 2 days of exposure to TCDD (1, 10 or 100 nM) and after 6 days recovery time without exposure. Each column represents mean ± SE of 300 tail moments in 3 independent experiments.

The Comet assay, in contrast to the MN assay, revealed that the stability of the genome was compromised in TCDD-exposed cells already at 2 days. When pretreated cells were challenged with menadione, the resulting DNA damage was increased in TCDD-pretreated cells (Fig. 2a). The overall effect of TCDD was significant at p = 0.0009, and the post tests showed a significant trend (p = 0.003) and that all TCDD groups were significantly (p<0.01) different from the menadione-only exposed group. The effect of menadione alone was significant at p<0.0001. Consistently with the micronucleus data, the TCDD effect showed a flat dose-response relationship between 1 and 100 nM. Interestingly, the increased sensitivity to menadione was not found if the cells were allowed to recover from TCDD for 6 days (Fig. 2b). The level of DNA strand breaks was even slightly (nonsignificantly) lower in the TCDD-exposed cells than in the menadione-only exposed cells at 0 and 15 min after the menadione treatment. It is important to note that the effect of TCDD was observable only as an increased sensitivity to menadione. TCDD alone, without menadione treatment, had no effect in the Comet assay (Fig. 3).

The PCR array method used for gene expression analysis showed very high reproducibility. The number of genes showing ≥2-fold changes was 3 in two independent comparisons of unexposed control samples and 2 in one such comparison. For ≥1.5-fold changes, the same comparisons yielded 8, 5 or 7 differentially expressed genes. One gene (Tnf) showed ≥2-fold differences in all three comparisons, and one (Serpinb2) in two of the three comparisons. These genes had very low expression levels and the comparison of their expression is therefore inaccurate.

**Figure 4:**
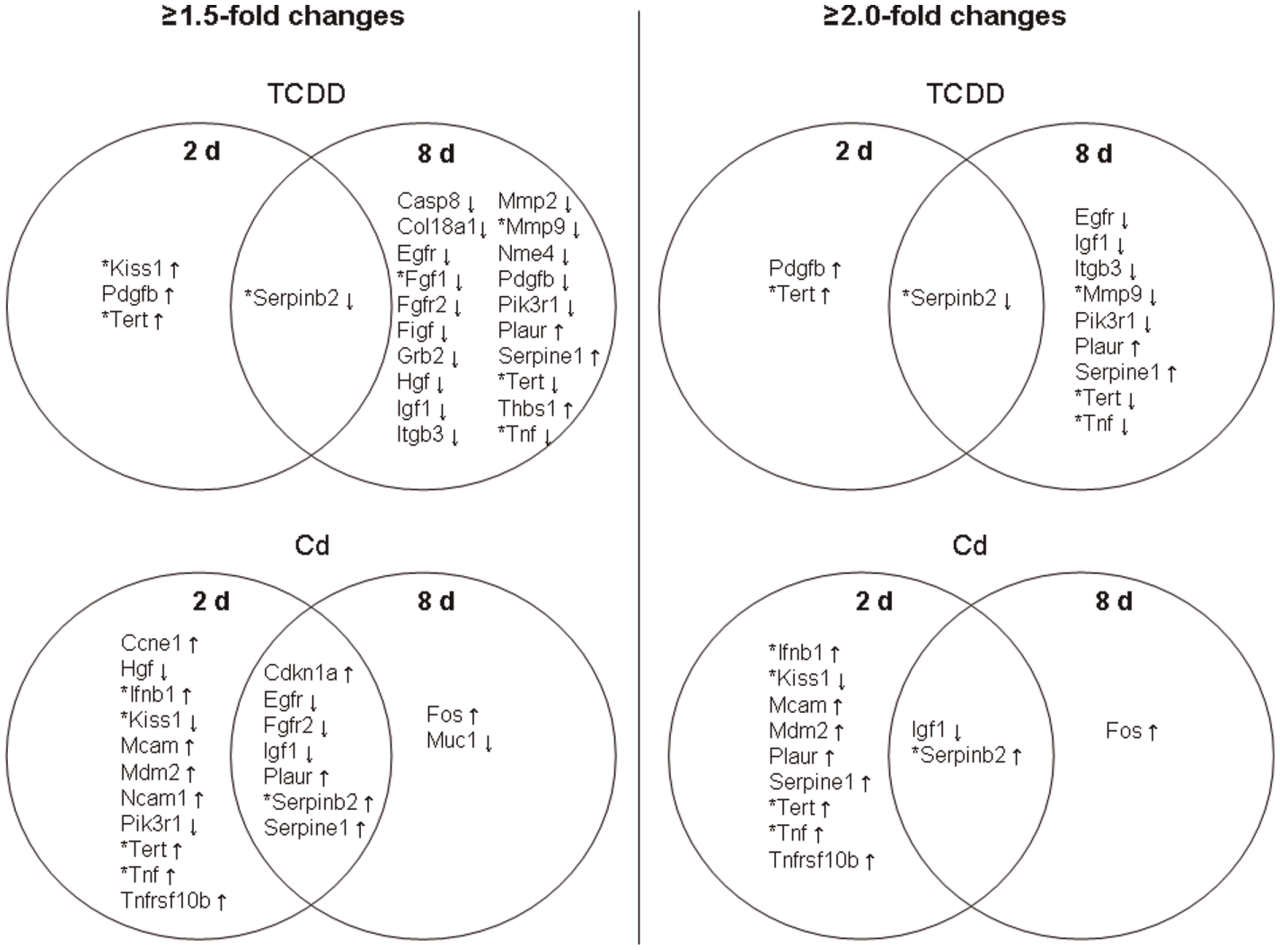
Venn diagrams showing gene expression changes. Direct and delayed alterations in expression of genes in the mouse CancerFinder PCR array are shown after 2 days treatment with 10 nM TCDD or 1 µM cadmium and after further culture without exposure. Both ≥1.5-fold (left panel) and ≥2.0-fold (right panel) up- or downregulations are shown. Asterisk (*) indicates a low level of expression (Ct>30) and therefore less reproducible measurement.

The gene expression data showed a pattern similar to the MN data indicating predominantly a delayed response to TCDD, but immediate response to cadmium (Fig. 4). With the threshold of fold changes set at ≥2.0, TCDD changed the expression of only 3 genes (2 of which had low expression levels and therefore tended to show relatively high variation) immediately after the 2-day exposure, but 10 genes were affected after 6 days of further culture without exposure. Only one affected gene was common at both time points. The delayed effects were mainly downregulations (8 down- and 2 upregulations), while 2 out of 3 of the immediate effects were upregulations.

Most of the TCDD-induced delayed changes occurred in genes involved in angiogenesis as well as invasion and metastasis (Table 1). When the threshold of 1.5 was used for fold changes, the number of differentially expresses genes was higher, but the pattern of responses was similar to that seen with the threshold of 2.0 (Fig. 4).

**Table 1:** Genes and gene groups in Mouse Cancer PathwayFinder PCR array.

Functional gene group	Genes
Cell cycle control and DNA damage repair	Atm, Brca1, Ccnd1, Ccne1, Cdc25a, Cdk2, Cdk4, Cdkn1a, Cdkn2a, Chek2, E2f1, Mdm2, Pten, Rb1, Trp53
Apoptosis and cell senescence	Apaf1, Bad, Bax, Bcl2, Bcl2l1, Birc5, Casp8, Cflar, Fas, Tert, Tnfrsf1a, Tnfrsf10b
Signal transduction molecules and transcription factors	Akt1, Akt2, Ctnnb1, Ets2, Fos, Grb2, Jun, Map2k1, Myc, Nfkb1, Nfkbia, Pik3r1, Raf1
Adhesion	Cdh1, Itga2, Itga3, Itga4, Itgav, Itgb1, Itgb3, Mcam, Ncam1
Angiogenesis	Angpt1, Col18a1, Egfr, Fgf1, Fgfr2, Figf, Hgf, Ifnb1, Igf1, Pdgfa, Pdgfb, Tek, Tgfb1, Tgfbr1, Thbs1, Tnf, Vegfa, Vegfb, Vegfc
Invasion and metastasis	Kiss1, Met, Mmp2, Mmp9, Mta1, Mta2, Muc1, Nme4, Plau, Plaur, S100a4, Serpinb2, Serpine1, Syk, Timp1, Twist1

### Discussion

TCDD is considered a nongenotoxic carcinogen (also termed epigenetic or non-DNA reactive carcinogen), as it does not appear to cause direct genetic damage [17], [18]. In rats, increased frequencies of micronucleated erythrocytes were observed only after long-term exposure to very high dose levels that caused overt toxicity, suggesting that induction of MN does not represent a specific genotoxic effect but rather a secondary response [7]. The findings of the present study are consistent with lack of direct genotoxic effects, as no increase in MN was observed immediately after two days of exposure. However, a delayed increase of MN was observed several cell generations later in the progeny of TCDD-exposed cells. Delayed increase in MN was also reported in two highly TCDD-exposed women several months after intoxication, when TCDD levels had already decreased [19]. To the best of our knowledge the present study is the first controlled experimental study showing delayed genetic damage as a result of TCDD exposure. This finding indicates that, although TCDD does not cause direct genotoxicity, it induces a state in which the likelihood of later genetic changes is increased in the progeny of the exposed cells, i.e., genomic instability. The finding is potentially important for understanding GI in general, as it shows that the initiating event does not need to be an extensive DNA damage, such as double-strand breaks induced by ionizing radiation.

The dose-response relationship of the observed TCDD-induced effects is interesting: both the delayed increase in MN and the increased sensitivity to menadione showed a flat dose-response. This finding is consistent with dose-response data reported for ionizing radiation-induced GI, i.e., a threshold at relatively low doses and a plateau above it [20], [21]. As no immediate genotoxicity was observed, the signal that transmits TCDD-induced GI to the later cell generations must be epigenetic by its nature. This is consistent with the current understanding that GI induced by ionizing radiation is inherited to next cell generations in an epigenetic fashion [22]–[25].

The gene expression results were consistent with the MN data, suggesting that also TCDD-induced alterations in expression of cell transformation and tumorigenesis related genes are delayed. Although 3 (or 4 with the 1.5-fold threshold) genes were affected immediately after exposure, the majority of changes were observed at 8 days. This pattern of altered gene expression is completely different from that of cadmium exposure, which caused more changes immediately after exposure than at 8 days (with most of the genes affected at 8 days being the same as those that were affected at 2 days). The gene expression changes also differ from those observed after exposure to ionizing radiation, which causes both immediate and delayed changes in gene expression [26]. The delayed nature of the changes therefore seems to be a special characteristic for TCDD. In the present study, only a limited number of cancer-related genes were measured, which should be taken into account in interpreting the low number of gene expression changes observed immediately after exposure. In a microarray study with human hepatoma HepG2 cells exposed to 10 nM TCDD, 310 out of 5686 known genes were altered by a factor of at least 2.1 immediately after exposure [27], the proportion of affected genes being close to that observed in the present study (3 out of 84). The findings of Puga et al. indicated a complex response involving multiple cellular processes [27]. The increased number of changes observed at 8 days in the present study suggests that an even more complex pattern of responses develops in a delayed manner in the progeny of the exposed cells.

Overall, the micronucleus and gene expression data observed in the present study indicate that delayed effects consistent with GI can be induced by TCDD with very little biological changes observable immediately after exposure. The results from the Comet assay, however, showed that sensitivity to a DNA-damaging agent (menadione) was increased already directly after TCDD treatment. This finding might also provide clues to the mechanisms of the delayed effects observed: the delayed increase in MN might arise from increased sensitivity of the cells to intracellular oxidative stress or other causes of “spontaneous” DNA damage. In other words, the impaired response to DNA damage (observable as altered response to menadione) might be inherited to the progeny of TCDD-exposed cells. It is of interest, however, that no changes were observed in the expression of any of the 15 representative genes involved in DNA repair or cell cycle control either immediately after exposure or at 8 days. The regulation of DNA damage responses is, however, very complex, and it is possible that all relevant genes were not measured in this study. Another detail that complicates interpretation of the data is the fact that the increased sensitivity to menadione had disappeared (or even reversed) at 8 days. It seems clear that TCDD alters responses to DNA damage, but additional experiments are needed to characterize this phenomenon and to understand its mechanisms.

In conclusion, TCDD was shown to induce delayed increase of MN in mouse embryonal fibroblasts. Exposure to TCDD also caused increased sensitivity to induction of DNA damage by subsequent exposure to menadione. These findings indicate that TCDD is able to induce GI and that such instability is associated with impaired DNA damage response. These effects showed flat dose-response similar to that reported for radiation-induced GI. The effects of TCDD on expression of cancer-related genes were also mainly delayed. The present results from experiments with TCDD indicate that direct extensive DNA damage is not needed to initiate genomic instability. Next, methylation analyses are under way to find out if TCDD-induced GI is associated with altered DNA methylation.

## Materials and Methods

### Chemicals

TCDD was purchased from Ufa-Institute (Ufa, Russia) and was over 99% pure as assessed by gas chromatography-mass spectrometry. Cadmium chloride (Fluka, over 99% pure) was obtained from Sigma. Media, serum and other products for cell culture were purchased from Gibco (Invitrogen, Paisley, UK). Menadione was supplied by Sigma-Aldrich (Steinheim, Germany).

### Cell Culture and Treatments

Mouse embryonic fibroblasts (C3H10T1/2 clone 8) were purchased from American Type Culture Collection. The cells were grown in Basal Eagle Medium supplemented with 10% Fetal Bovine Serum, 2 mM L-glutamine and 100 U/ml penicillin & 100 µg/ml streptomycin at 37°C in a humidified atmosphere of 5% CO_2_ in air. For the gene expression analysis, cells were plated at the density of 5000 cells/cm^2^. On the next day, the medium was replaced with exposure medium containing 10 nM TCDD dissolved in DMSO, or DMSO vehicle alone, so that the final concentration of DMSO was only 0.1%. Cells were also exposed to 1 µM cadmium chloride. After 2 days the exposure medium was removed and cells comprising 2-d samples were harvested. For 8- or 15-d samples, the cells were subcultured and grown for 6 or 13 more days without exposure.

For MN analysis, ∼1900 cells/cm^2^ were seeded on Petri plates (for 2-d samples) or flasks (for 8-d samples) 24 h prior to exposure. In addition to the exposures used in the gene expression assays, cells were also exposed to 1 and 100 nM TCDD and etoposide (0.025 µg/µl), which was used as a positive control. Separate control groups with or without DMSO were used for TCDD exposures and cadmium exposures, respectively. After 2 days exposure, micronucleus analysis was performed for 2-d samples. For 8-d samples, cells were subcultured twice before MN analysis. At the last subculturing 2 days before the MN analysis, ∼4700 cells/cm^2^ were seeded on Petri plates.

For the Comet assay, ∼2300 cells/cm^2^ and 1200 cells/cm^2^ were seeded on Petri plates for 2- and 8-d samples, respectively. 24 h after subculturing, the cells were exposed to 1, 10, or 100 nM TCDD. After 2 days of exposure, TCDD was removed and 40 µM menadione was applied for 1 h (for 8-d samples, TCDD was replaced only with fresh medium). After menadione treatment, fresh medium was applied on the cell cultures. Cells were allowed to repair menadione-induced DNA damage for 0, 15, or 30 min. The time dependent decrease in Olive Tail Moments (OTM) was used as a measure of DNA repair. After repair period, cells were detached from plates by incubating cultures 5 min in 1 ml of 0.25% trypsin (Invitrogen, Carlsbad, CA, USA) in 0.02% EDTA in PBS (w/o Ca^2+^, Mg^2+^) for 5 min, after which trypsin was inactivated by adding 2 ml of fresh 37°C medium. Cell suspension was transferred to 15 ml tubes, centrifuged and used for the Comet assay. For 8-d samples, the cells were subcultured (1∶10) one day after the end of TCDD exposure; otherwise the protocol was the same.

### Cell Proliferation Assay

For the proliferation test, cells were grown on 24-well plates. Cell proliferation was determined by colorimetric assay using Cell Proliferation Reagent WST-1 (Roche, Mannheim, Germany). First, medium was removed and WST-1 reagent added to wells. Cells were incubated for 1 h. After shaking the cell plate for 1 min, the media were transferred into 96-well-plate. The absorbance of the samples was measured using a plate reader at 450 nm (Labsystems iEMS Reader MF, Ascent Software version 2.4.1).

### Micronucleus Analysis

Micronucleus frequency of cells was analysed by a flow cytometry based assay as described by Luukkonen et al. [14], with minor changes. In brief, cells were stained with ethidium monoazide bromide (EMA), photoactivated with visible light (light bulb) and the nuclei stained with SYTOX Green dye. The principle of this method is staining first the nuclei of dying cells (i.e. cells with damaged cell membrane) with EMA, lysing the cells, staining the nuclei with SYTOX Green and assorting the nuclei by flow cytometry [28].

In brief, cell cultures were incubated on ice for 20 min, 1.5 ml of 8.5 µl/ml EMA-solution (4°C) was added and the stain was light activated under light bulb for 30 min. Cell cultures were washed once with 1.5 ml FBS in PBS (w/o Ca^2+^ and Mg^2+^, 4°C), depending on the observed cell density on plates 0.5–0.7 ml of lysis 1-solution (0.3 µl IGEPAL/ml, 0.584 mg NaCl/ml, 0.5 mg RNase A/ml, 1 mg sodium citrate/ml, and 0.4 µM SYTOX Green in MilliQ-water, 4°C) was added and plates were incubated light-protected at 37°C for 1 h. After incubation, 0.5–0.7 ml of lysis 2-solution (15 mg citric acid/ml, 85.6 mg sucrose/ml, 0.4 µM SYTOX Green, and 1 drop of 6 µm fluorescent beads, 20°C) was added and cell cultures were incubated light-protected at room temperature for 30 min. Finally, a flow cytometer (Becton Dickinson FACSCalibur, Becton Dickinson, San Jose, CA) was used in MN analyses according to Bryce et al. [28] and data were analyzed by CellQuest Software (v.3.3 Becton Dickinson, San Jose, CA). Data were collected from 4 separate experiments.

### DNA Strand Breaks and DNA Repair

DNA damage and repair were measured by the Comet assay (single cell gel electrophoresis). An alkaline version of the method (pH>13) detecting both DNA single-strand breaks (SSB) and DNA double-strand breaks (DSB) was used as described earlier [29]. In this method, cells are mixed with agarose, spread on glass microscope slide and lysed by lysis buffer leaving only nuclei on the slide. During electrophoresis, broken DNA fragments migrate away from the nucleus forming a tail that resembles a comet. The size, shape and fragment content of the tail reflect the extent of the DNA damage.

The analysis was performed as described by Luukkonen et al. [14]. Minor modifications on suspension volumes and electrophoresis conditions were: cell pellet was suspended to 350 µl (for 2-d samples) or 1500 µl (for 8-d samples) of cold PBS and 15 µl (∼1.7×10^4^ cells) of the suspension was embedded in 75 µl of melted 0.5% LMP (low melting point) -agarose at 37°C. Unwinding time for electrophoresis was 15 min and electrophoresis was run for 15 min. Data was collected from 4 separate experiments.

### PCR Array Analysis

RNA was isolated from pelleted cells using RNeasy Mini Kit and RNAse free DNase (Qiagen, Hilden, Germany). The concentration of RNA was determined using Nano Drop (Thermo Scientific, Wilmington, DE, USA). 1 µg of RNA was generated into cDNA using RT^2^ First Strand Kit (SABiosciences, a Qiagen Company). cDNA samples were mixed with RT^2^ qPCR Master Mix (SABiosciences) and distributed in every well on PCR array plate (Mouse Cancer PathwayFinder RT^2^ Profiler PCR array by SABiosciences). This PCR Array profiled the expression of 84 genes representative of the six biological pathways involved in transformation and tumorigenesis (Table 1) and has been reported to exhibit good reproducibility and highly comparable results in gene expression measurements with high-density microarrays [30]. The array also contained controls for RT reaction and PCR reaction as well as a genomic DNA control. Applied Biosystems 7000 Real-Time PCR System (Applied Biosystems) was used to determine Ct-values of each well. The fold-changes in Ct-values were calculated using SABiosciences’ web-based data analysis program. Results represent mean of two independent experiments and two individual PCR arrays per experiment.

### Statistical Analysis

The MN and Comet assay data were analyzed with repeated measures ANOVA (TCDD data), paired t-test (cadmium data) or unpaired t-test (effect of menadione in the Comet assay). Test for linear trend and Dunnett’s test (comparison of all treated groups with controls) were used as post-test in ANOVA. In the Comet assay data, cells exposed to menadione only were used as the comparison group for the effect of TCDD. The GraphPad Prism 4.03 software (GraphPad Software, Inc., La Jolla, California) was used for the statistical analyses.
